# Primary Non-Germinal Center-Type Large B-Cell Lymphoma Involving the Thoracic Epidural Space, Cauda Equina, and Filum Terminal: Diagnosis and Treatment Using Biportal Endoscopic Spine Surgery—A Case Report and Literature Review

**DOI:** 10.3390/reports9010061

**Published:** 2026-02-13

**Authors:** Nan-Fu Chen, Chien-Yu Ou

**Affiliations:** Department of Surgery, Kaohsiung Armed Forces General Hospital, Kaohsiung 802, Taiwan; chen06688@gmail.com

**Keywords:** unilateral biportal endoscopy, primary lymphoma, thoracic, cauda equina, B-cell lymphoma

## Abstract

**Background and Clinical Significance**: We report a rare case of a 66-year-old male with malignant non-germinal center-type large B-cell lymphoma involving the thoracic epidural, cauda equina, and filum terminal simultaneously. **Case Presentation**: The patient complained of back pain, rapid progressive numbness, and motor palsy in both legs in one month. Neurological examination revealed grade 2 muscle power in both lower limbs, hypesthesia below the T8 dermatome, and bladder and bowel dysfunctions. Magnetic resonance imaging (MRI) with contrast showed a well-defined extradural lesion extending from the T7 to T9 level, with severe spinal cord compression. Additionally, it revealed enlargement of the cauda equina occupying the extradural space from the L1-S1 level. The lesion appeared isointense on T1, mildly hyperintense on T2-weighted images, and exhibited homogeneous enhancement on post-contrast images. To relieve the patient’s spinal cord compression as soon as possible and allow the patient to recover quickly after surgery, we performed unilateral biportal endoscopy (UBE) to completely remove the T7-9 epidural lesion. The immunohistochemical assessment confirmed a histological diagnosis of diffuse large B-cell lymphoma, a non-germinal center type. The patient received radiotherapy to the thoracic and lumbosacral areas (50 Gy) and chemotherapy with six cycles of rituximab, cyclophosphamide, doxorubicin, vincristine, and prednisolone (R-CHOP) after surgery. Follow-up positron emission tomography (PET) scan and MRI performed 4 months after surgery revealed complete remission of the lesion. The patient was able to walk using a walker after therapy. **Conclusions**: UBE is a favorable option for selected patients requiring immediate chemotherapy or radiotherapy owing to its reduced tissue trauma compared to traditional open surgery.

## 1. Introduction and Clinical Significance

Diffuse large B-cell lymphoma (DLBCL) is the most common type of non-Hodgkin lymphoma, representing 30–40% of non-Hodgkin lymphoma cases worldwide. Approximately 90% of the primary central nervous system lymphomas (PCNSLs) are diffuse large B-cell lymphomas [1,2]. However, primary diffuse large B-cell lymphoma of the central nervous system (CNS) accounts for 0.5–3% of primary CNS tumors and ≤1% of all lymphomas [3,4,5]. Diffuse large B-cell lymphomas occurring in the spinal epidural space or cauda equina are rare. Primary spinal epidural diffuse large B-cell lymphoma accounts for only 1.8% of all diffuse large B-cell lymphomas [6]. Less than 1% of PCNSLs occur in the spinal cord [7]. Moreover, among spinal cord lymphomas, cauda equina lesions are even rarer, and only sporadic cases have been reported [8,9]. Although surgical decompression can prevent further deterioration of neurological function in such cases, it does not fully address the patient’s underlying condition. The most important factor is to ensure that patients receive chemotherapy and radiotherapy as soon as possible.

We present a rare case in which the lesions occurred simultaneously in the thoracic epidural space, cauda equina, and filum terminal, causing neurological function to deteriorate in a short period. Due to the patient’s distinctive preoperative MRI images, we prioritized lymphoma at the top of the differential diagnosis. To ensure that the patient receives chemotherapy and radiotherapy as soon as possible after surgery, we chose the unilateral biportal endoscopy (UBE) surgical technique to achieve thoracic spinal cord decompression and obtain a pathological diagnosis simultaneously. After receiving chemotherapy and radiotherapy, his positron emission tomography (PET) scan revealed complete tumor remission, and his neurological function recovered quickly 4 months after surgery. To the best of our knowledge, this is the first reported case of primary spinal DLBCL invading the thoracic epidural space, cauda equina, and filum terminal simultaneously. It is also the first case of primary spinal DLBCL treated with UBE surgery.

## 2. Case Presentation

**Figure 1:**
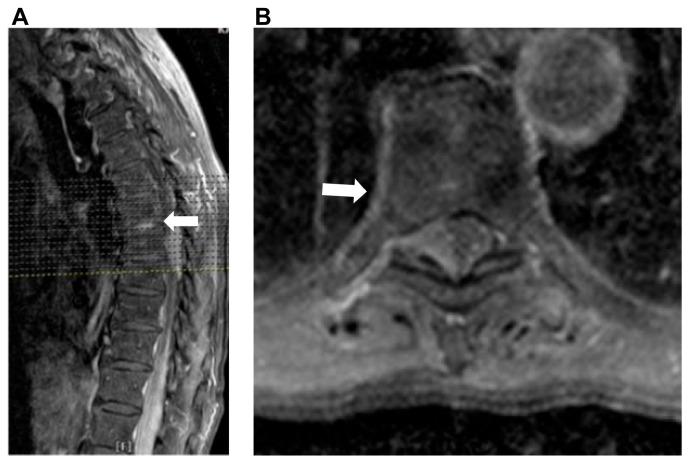
MRI of thoracic spine: (**A**) sagittal contrast-enhanced image showing a well-defined epidural soft tissue mass at the T7-T9 level (arrow); (**B**) axial contrast-enhanced image at the T8 level showing a right-sided, homogenously enhanced extradural lesion with spinal cord compression (arrow).

A 66-year-old male presented with a history of back pain, rapid progressive numbness, and motor palsy in both legs in one month. For the last 7 days, he was unable to walk owing to the increased severity of his symptoms, and he was admitted to our hospital in a wheelchair. He also complained of difficulties in defecation and urination. His symptoms could not be alleviated, even with the administration of analgesics. Neurological examination revealed marked palsy of both lower limbs with a score of 2, hypesthesia below the T8 dermatome, and a positive Babinski sign in both feet. Due to difficulty in urination, a foley catheter was placed in another hospital before visiting our clinic. Magnetic resonance imaging (MRI) of the thoracic spine revealed a well-defined right-sided extradural mass lesion that was 5.7 × 2.1 × 1.8 cm in size, extending from the T7 to T9 vertebral level, causing severe compression and displacement of the spinal cord to left side (Figure 1A,B).

**Figure 2:**
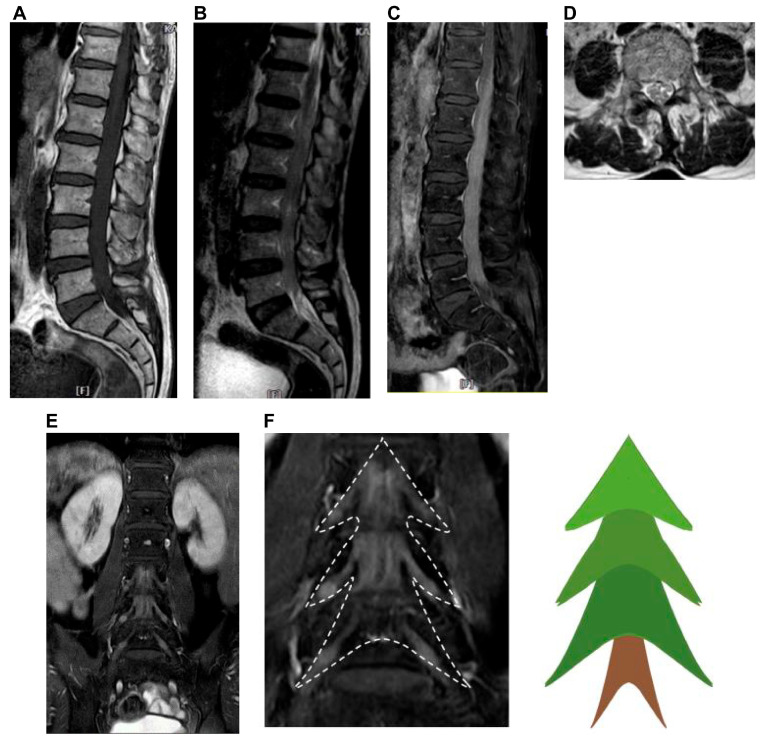
MRI of Lumbar spine. (**A**) Sagittal T1-weighted image showing homogeneous iso- to hypointensity compared to the conus. (**B**) Sagittal T2-weighted image showing heterogeneous iso- to hyperintensity compared to the conus, with no cerebrospinal fluid (CSF) signal. (**C**) Sagittal enhanced T1-weighted image showing diffuse enhancement of the cauda equina roots from the level of the L1 to S1 vertebra. (**D**) Axial T2-weighted image showing enlargement of cauda equina roots at the level of the L3 body. (**E**) Coronal enhanced T1-weighted image showing enhancement of cauda equina and the nerve roots. (**F**) The enhanced cauda equina and nerve roots resemble a Christmas tree-like appearance.

MRI of the lumbar spine revealed enlargement of the cauda equina and filum terminal, occupying the dural sac from the L1 to S1 level (Figure 2A–C).

The lesion showed homogeneous iso- to hypointensity on T1 and heterogeneous iso- to hyperintensity on T2-weighted images, with homogeneous enhancement on post-contrast images. Additionally, there was diffuse enhancement of the cauda equina with thickening of the filum terminalis and nerve roots (Figure 2D) which resembled a Christmas tree in coronal view (Figure 2E,F). Extensive work-up for malignancy, including tumor markers and brain and abdominal computed tomography findings, was negative. We also performed a positron emission tomography–computed tomography (PET/CT) scan of the patient and found a highly metabolic lesion in the spinal area (T12–S1), which corresponded with the MRI findings. No lesions were found in any other locations on (PET/CT) scan. The possible differential diagnoses based on these examinations included lymphoma, granuloma, and metastatic tumors; however, lymphoma was ranked first in the differential diagnosis because of its unique MRI findings.

**Figure 3:**
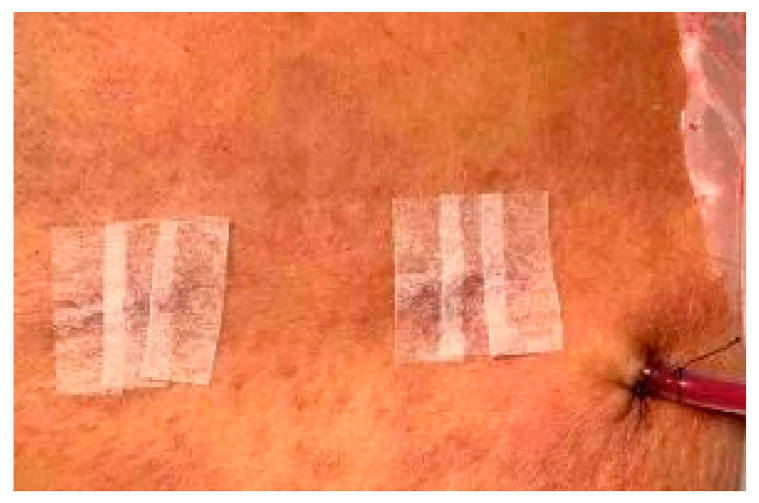
Surgical wounds.

The patient’s neurological function deteriorated rapidly, and pain in both lower limbs could no longer be relieved by morphine. To improve the patient’s neurological function and neuropathic pain and to allow the patient to receive radiotherapy and chemotherapy as early as possible after surgery, we used the UBE surgical technique to remove the thoracic extradural tumor and perform complete spinal cord decompression. Only two small skin incisions were made during surgery (Figure 3).

**Figure 4:**
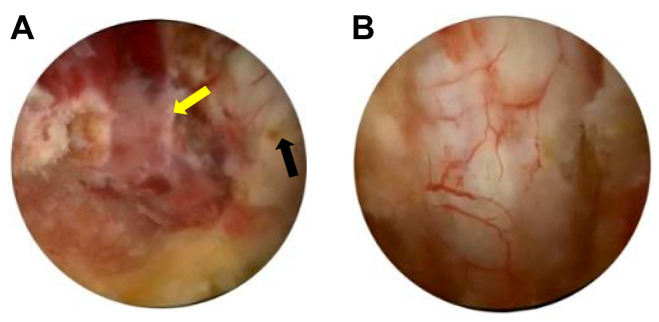
Intraoperative endoscopic pictures. (**A**) The thoracic epidural tumor (yellow arrow) appears gray, soft, friable, and hypervascular, causing compression of the dural sac (black) to the left side. (**B**) Dural expansion was observed following decompression.

Intra-operatively, the lesion was located on the right side of the epidural space, extending from the T7 to the T9 vertebral bodies. The lesion appeared gray, soft, friable, and hypervascularized (Figure 4A).

After removal of the extradural tumor, adequate nerve decompression was immediately confirmed by normal pulsation of the dura under direct endoscopic vision (Figure 4B).

**Figure 5:**
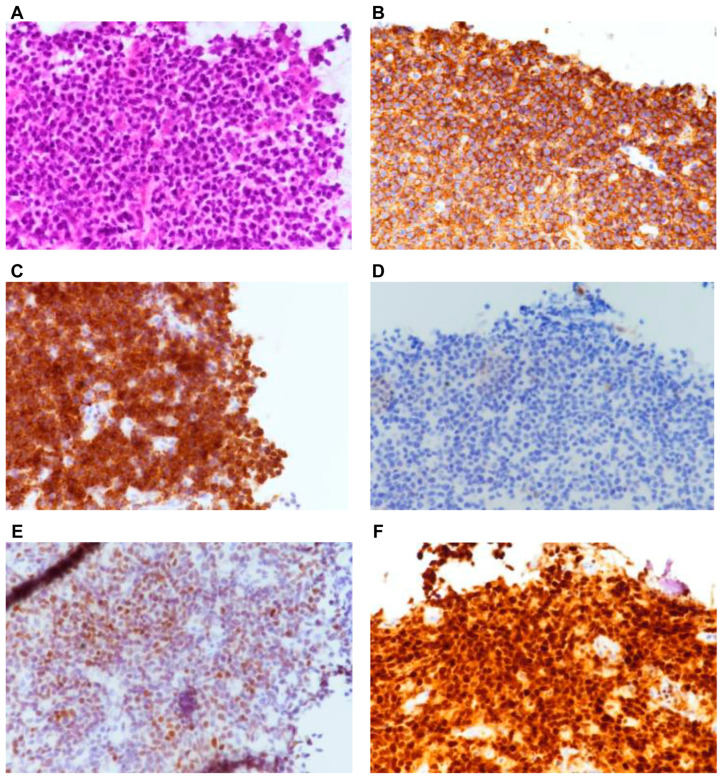
(**A**) Hematoxylin and eosin (magnification, ×400) staining revealed a pleomorphic population of mononuclear cells with irregular large nuclei, little cytoplasm, and mitotic figures. (**B**) The cells were positive for the CD20 B cell marker (magnification, ×400). (**C**) BCL-2 immuno-histochemical staining was positive (magnification, ×400). (**D**) CD 10 immuno-histochemical staining was negative (magnification, ×400). (**E**) BCL-6 labeling was more than 30% positive (magnification, ×400). (**F**) MUM-1 immuno-histochemical staining revealed strong positivity. CD, cluster of differentiation; BCL2, B-cell lymphoma 2; MUM-1, multiple myeloma oncogene 1 (magnification, ×400).

On histopathological examination, the mass was described as an ill-defined infiltrative lesion composed of heavy lymphoid infiltration with variable-sized lymphoid follicle formations (Figure 5A). Immunohistochemical staining revealed that the cells were positive for CD20 and BCL-2 (Figure 5B,C). However, they were negative for CD10 (Figure 5D). BCL-6 labeling was greater than 30%, and MUM-1 showed strong positivity (Figure 5E,F). Therefore, the pathology was diagnosed as diffuse large B-cell lymphoma (DLBCL), consistent with the non-germinal center type [6,10]. The positive expression of BCL-2 indicated the tumor’s aggressiveness.

**Figure 6:**
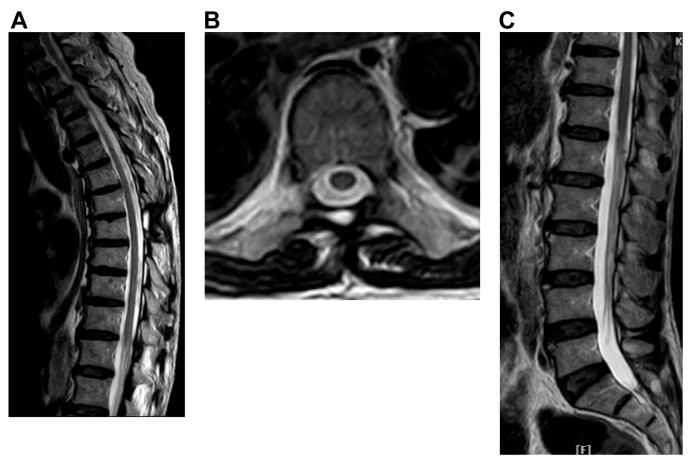
Post-op MRI 4 months after induction of chemotherapy. (**A**) T2-weighted sagittal image of thoracic spine showing no spinal cord compression by the tumor. (**B**) T2-weighted axial image of the thoracic region at the T8 level showing full expansion of the dural sac without tumor compression. (**C**) T2-weighted sagittal images of the cauda equina showing reappearance of CSF signals in the dural sac.

After surgery, the patient was referred to an oncologist and received six cycles of chemotherapy with rituximab, cyclophosphamide, doxorubicin, vincristine, and prednisolone (R-CHOP). Palliative radiotherapy was also administered to the surgical sites and lumbar region. MR images of the thoracic and lumbar spines did not show any enhancement in the thoracic spine and cauda equina 4 months after the completion of chemotherapy (Figure 6). Follow-up PET–computed tomography 4 months after chemotherapy revealed no abnormal metabolic activity. Motor function was progressively restored following rehabilitation, and the patient was able to walk with a walker.

## 3. Discussion

Primary central nervous system lymphoma (PCNSL) is an aggressive lymphoma confined to the brain, spinal cord, leptomeninges, CSF, and the vitreoretinal compartments of the eye [11]. PCNSL accounts for 4–6% of all malignant lymphomas [12]. However, DLBCL comprises the majority (90–95%) of PCNSL [13]. Primary DLBCL of the CNS constitutes only 0.5% of all primary CNS tumors [3,4,5]. Primary spinal epidural DLBCL accounts for 1.8% of all diffuse large B-cell lymphomas [6]. For primary spinal epidural lymphoma, the thoracic spine is the most commonly involved site, followed by the lumbosacral and cervical spine [14].

Neurolymphomatosis (NL) is characterized by the infiltration of malignant lymphoma cells into peripheral nerves, spinal nerve roots, plexuses, or cranial nerves [15,16,17]. Malignant lymphoma arising in the cauda equina and filum terminale is included in the entity of neurolymphomatosis. NL is a rare malignant lymphoma, accounting for only 0.2% of non-Hodgkin ‘s lymphoma cases [18]. NL can be categorized into two types: primary and secondary. Primary NL is defined as the initial presentation of a hematological malignancy, whereas secondary NL occurs when the condition develops as a site of relapse or progression from a previously diagnosed lymphoma or leukemia [15]. Additionally, primary NL is less common than secondary NL [19]. Primary cauda equina lymphoma (PCEL) accounts for less than 1% of all neurolymphomatosis cases [18]. According to statistics in the literature, less than 1% of PCNSLs occur in the spinal cord [7], and lymphoma involving the cauda equina is even rarer.

The incidence of primary diffuse large B-cell lymphoma (PDLBCL) developing alone in the thoracic spinal epidural space, cauda equina, or filum terminal is very low, and the incidence of developing PDLBCL in these areas simultaneously is even lower.

Lymphoma is uncommon in the epidural space of the spine. Therefore, it is rarely listed as a primary differential diagnosis. However, in this case, the tumor also showed infiltration of the cauda equina, filum terminale, and numerous nerve roots, which is rare in metastatic tumors. Therefore, lymphoma was considered a more likely disease in the differential. Primary cauda equina and filum terminale lymphoma as primary NL is an extremely rare condition [16,20,21]. A previous study pointed out that diffuse large B-cell type lymphoma makes up approximately 82.6% of reported cases of cauda equina lymphoma (CEL) [20]. The MRI of cauda equina and filum terminal lymphoma is also very unique and rare. The most common MR findings of CEL are enhanced swelling or enlargement of the cauda equina and nerve roots in axial views (Figure 2D) and loss of CSF signal on T2WI sagittal views (Figure 2B) [20,21,22]. In addition, the contrast-enhanced cauda equina and nerve roots appear to resemble a Christmas tree in coronal views, creating a unique image (Figure 2E,F). This particular image has not been reported in previous literature.

Rapid neurological deterioration in this patient with loss of bladder function and paraplegia occurred over the last few days, which was caused by simultaneous tumor compression of the thoracic spinal cord and cauda equina. Although lymphomas are very chemo- and radiosensitive tumors, treatment with radiotherapy alone, chemotherapy alone, or radiation therapy combined with chemotherapy can be effective for managing PDLBCL of the spine [23,24,25]. However, surgical decompression remains essential to obtain a correct diagnosis in such cases. Several studies have recommended that patients with spinal cord or cauda equina compression due to lymphoma require surgical management to achieve nerve decompression and to obtain a correct histological diagnosis. Early surgery in conjunction with aggressive chemotherapy and radiotherapy is associated with good functional outcomes [26,27,28]. In addition, a previous study demonstrated that the functional recovery of patients with spinal cord compression due to primary spinal epidural lymphoma is relatively better than that of patients with metastatic carcinoma [29,30]. For these reasons, this patient required immediate decompressive surgery to prevent the progression to complete spinal cord injury. However, in this patient, the tumor not only invaded the thoracic spine but also the cauda equina and numerous nerve roots. The primary goal is to obtain a tissue diagnosis while ensuring the patient can receive subsequent radiotherapy and chemotherapy as soon as possible. Although a simple tissue biopsy can provide an immediate tissue diagnosis, it does not quickly improve thoracic spinal cord compression and is not an effective treatment option. Surgical decompression can provide tissue diagnosis as well as an opportunity for decompression of the spinal cord and restoration of the patient’s neurological function. However, traditional open surgery involves large surgical wounds, increased tissue damage, and post-operative pain. Lymphoma mostly occurs in elderly and immunocompromised patients [31,32]. Such patients usually have poor immune function and malnutrition. If such patients undergo traditional open surgery, they may face problems such as poor wound healing, wound infection, and bleeding after surgery. This delays the subsequent radiotherapy and chemotherapy. To relieve compression of the thoracic spinal cord, shorten the postoperative wound recovery time, and allow patients to receive radiotherapy and chemotherapy as soon as possible, we used the UBE surgical technique. UBE is a newly developed spinal endoscopic technique that was first reported by Antoni et al. in 1996 [33]. Only two small portals were made during this surgery: an endoscopic portal and a working portal (Figure 3). This minimally invasive spine surgery has the advantages of a small skin incision, less blood loss, and a shorter hospital stay and has been used in treating lumbar disc herniation and lumbar spinal stenosis with good clinical outcomes [34,35]. With the ongoing advancement and widespread adoption of this surgical technique, the UBE technique has also been used in spine fusion surgery [36,37]. Additionally, this surgical technique has been employed to treat cervical disc herniation and myelopathy [38,39], as well as ossification of the posterior longitudinal ligament and ligamentum flavum in thoracic spines [40,41]. Although there have been sporadic case reports in the literature using UBE technique to treat spinal tumors [42,43], to the best of our knowledge, this study was the first to apply the UBE techniques to treat thoracic epidural large B-cell lymphoma. The endoscopic view, facilitated by continuous normal saline irrigation, provides superior surgical field clarity compared to traditional microscopy, allowing for more precise hemostasis. The patient’s neuropathic pain significantly improved after surgery. Owing to the small skin incision and reduced tissue trauma after biportal endoscopic surgery, the surgical wound healed well without infection. He received palliative radiotherapy followed by R-CHOP chemotherapy earlier, which resulted in complete remission of the tumor and quicker recovery of neurological function. This case presentation has several limitations. First, the manuscript presents a single patient case, making it difficult to draw definitive conclusions without larger cohort studies. Additionally, the study primarily focuses on the use of the UBE surgical technique without direct comparison to other treatment methods, limiting the ability to evaluate its relative efficacy. Lastly, since endoscopic surgery is performed in a fluid-filled environment, collecting all resected pathological tissues remains a challenge. Future research should focus on conducting multicenter studies with larger patient populations to evaluate the efficacy and safety of minimally invasive surgical techniques like UBE in managing primary CNS lymphomas involving complex spinal involvement. A final limitation is the brief four-month follow-up period for a disease characterized by high recurrence rates; however, the patient remains under active clinical surveillance.

## 4. Conclusions

Primary CNS large B-cell lymphomas are rare, with even more infrequent cases involving simultaneous invasion of the thoracic epidural space, cauda equina, and filum terminale, which can lead to rapid neurological decline. Early diagnosis and treatment are essential to prevent further neurological decline in such cases. Special and unique MRI images can serve as the basis for early diagnosis. Emergency neural decompression and early tissue diagnosis are crucial for patients presenting with severe symptoms of spinal cord compression. From this case, we found that UBE surgery offers a favorable option for selected patients requiring immediate chemotherapy or radiotherapy due to its reduced tissue trauma compared to traditional open surgery.

## Data Availability

The original contributions presented in this study are included in the article. Further inquiries can be directed to the corresponding author.

## Abbreviations

The following abbreviations are used in this manuscript:

PCNSL	Primary central nervous system lymphoma
CSF	Cerebrospinal fluid
DLBCL	Diffuse large B-cell lymphoma
PDLBCL	Primary diffuse large B-cell lymphoma
NL	Neurolymphomatosis
PCEL	Primary cauda equina lymphoma
UBE	Uniportal biportal endoscopic
R-CHOP	Chemotherapy regimen (rituximab, cyclophosphamide, doxorubicin, vincristine, and prednisone)

## References

[1] Li S., Young K.H., Medeiros L.J. (2018). Diffuse large B-cell lymphoma. Pathology.

[2] Fakhouri F., Shoumal N., Obeid B., Alkhoder A. (2020). Primary Diffuse Large B-Cell Non-Hodgkin’s Lymphoma of the Thoracic Spine Presented Initially as an Epigastric Pain. Asian J. Neurosurg..

[3] Chen D., Gu W., Li W., Liu X., Yang X. (2016). Primary diffuse large B-cell lymphoma of the central nervous system: A case report and literature review. Oncol. Lett..

[4] Gerstner E.R., Batchelor T.T. (2010). Primary central nervous system lymphoma. Arch. Neurol..

[5] Pascoa Pinheiro J., Rato J., Rebelo O., Costa G. (2020). Primary spinal epidural lymphoma: A rare entity with an ambiguous management. BMJ Case Rep..

[6] Wada N., Kohara M., Ikeda J., Hori Y., Fujita S., Okada M., Ogawa H., Sugiyama H., Fukuhara S., Kanamaru A. (2010). Diffuse large B-cell lymphoma in the spinal epidural space: A study of the Osaka Lymphoma Study Group. Pathol. Res. Pract..

[7] Hochberg F.H., Miller D.C. (1988). Primary central nervous system lymphoma. J. Neurosurg..

[8] Nakashima H., Imagama S., Ito Z., Ando K., Kobayashi K., Ukai J., Muramoto A., Shinjyo R., Matsumoto T., Yamauchi I. (2014). Primary cauda equina lymphoma: Case report and literature review. Nagoya J. Med. Sci..

[9] Broen M., Draak T., Riedl R.G., Weber W.E. (2014). Diffuse large B-cell lymphoma of the cauda equina. BMJ Case Rep..

[10] Hans C.P., Weisenburger D.D., Greiner T.C., Gascoyne R.D., Delabie J., Ott G., Müller-Hermelink H.K., Campo E., Braziel R.M., Jaffe E.S. (2004). Confirmation of the molecular classification of diffuse large B-cell lymphoma by immunohistochemistry using a tissue microarray. Blood.

[11] Mendez J.S., Ostrom Q.T., Gittleman H., Kruchko C., DeAngelis L.M., Barnholtz-Sloan J.S., Grommes C. (2018). The elderly left behind-changes in survival trends of primary central nervous system lymphoma over the past 4 decades. Neuro-Oncology.

[12] Deckert P.W., Kluin P.M., Ferry J.A., Louis D.N., Wiestler O.D., Cavenee W.K. (2016). Diffuse large B-cell lymphoma of the CNS. WHO Classification of Tumours of the Central Nervous System.

[13] Lauw M.I.S., Lucas C.G., Ohgami R.S., Wen K.W. (2020). Primary central nervous system lymphomas: A diagnostic overview of key histomorphologic, immunophenotypic, and genetic features. Diagnostics.

[14] Xiong L., Liao L.M., Ding J.W., Zhang Z.L., Liu A.W., Huang L. (2017). Clinicopathologic characteristics and prognostic factors for primary spinal epidural lymphoma: Report on 36 Chinese patients and review of the literature. BMC Cancer.

[15] Grisariu S., Avni B., Batchelor T.T., van den Bent M.J., Bokstein F., Schiff D., Kuittinen O., Chamberlain M.C., Roth P., Nemets A. (2010). Neurolymphomatosis: An International Primary CNS Lymphoma Collaborative Group report. Blood.

[16] Khong P., Pitham T., Owler B. (2008). Isolated neurolymphomatosis of the cauda equina and filum terminale: Case report. Spine.

[17] Baehring J.M., Batchelor T.T. (2012). Diagnosis and management of neurolymphomatosis. Cancer J..

[18] Baehring J.M., Damek D., Martin E.C., Betensky R.A., Hochberg F.H. (2003). Neurolymphomatosis. Neuro-Oncology.

[19] Lagarde S., Tabouret E., Matta M., Franques J., Attarian S., Pouget J., Maues De Paula A., Figarella-Branger D., Dory-Lautrec P., Chinot O. (2014). Primary neurolymphomatosis diagnosis and treatment: A retrospective study. J. Neurol. Sci..

[20] Suzuki K., Yasuda T., Hiraiwa T., Kanamori M., Kimura T., Kawaguchi Y. (2018). Primary cauda equina lymphoma diagnosed by nerve biopsy: A case report and literature review. Oncol. Lett..

[21] Kuhlman J.J., Alhaj Moustafa M., Gupta V., Jiang L., Tun H.W. (2021). Primary cauda equina lymphoma treated with CNS-centric approach: A case report and literature review. J. Blood Med..

[22] Kumar N., Dyck P.J. (2005). Hypertrophy of the nerve roots of the cauda equina as a paraneoplastic manifestation of lymphoma. Arch. Neurol..

[23] Ferreri A.J.M., Calimeri T., Cwynarski K., Dietrich J., Grommes C., Hoang-Xuan K., Hu L.S., Illerhaus G., Nayak L., Ponzoni M. (2023). Primary central nervous system lymphoma. Nat. Rev. Dis. Primers.

[24] Cao S., Fan B., Sun Q., Chen J., Song X., Yin W. (2023). Comparison of the effect of chemoradiotherapy and chemotherapy on the survival of patients with primary diffuse large B-cell lymphoma of the spine: A SEER-Based Study. World Neurosurg..

[25] Milgrom S.A., Pinnix C.C., Chi T.L., Vu T.H., Gunther J.R., Sheu T., Fowler N., Westin J.R., Nastoupil L.J., Oki Y. (2018). Radiation therapy as an effective salvage strategy for secondary CNS lymphoma. Int. J. Radiat. Oncol. Biol. Phys..

[26] Mally R., Sharma M., Khan S., Velho V. (2011). Primary lumbo-sacral spinal epidural non-Hodgkin’s lymphoma: A case report and review of literature. Asian Spine J..

[27] Monnard V., Sun A., Epelbaum R., Poortmans P., Miller R.C., Verschueren T., Scandolaro L., Villa S.B., Majno S.O., Ozsahin M. (2006). Primary spinal epidural lymphoma: Patients’ profile, outcome, and prognostic factors: A multicenter Rare Cancer Network study. Int. J. Radiat. Oncol. Biol. Phys..

[28] Cho J.H., Cho D.C., Sung J.K., Kim K.T. (2012). Primary malignant lymphoma in a spinal cord presenting as an epidural mass with myelopathy: A case report. Korean J. Spine.

[29] Tsukada T., Ohno T., Tsuji K., Kita K., Kobayashi T., Deguchi K., Shirakawa S. (1992). Primary epidural non-Hodgkin’s lymphoma in clinical stage IEA presenting with paraplegia and showing complete recovery after combination therapy. Intern. Med..

[30] Haddad P., Thaell J.F., Kiely J.M., Harrison E.G., Miller R.H. (1976). Lymphoma of the spinal extradural space. Cancer.

[31] Sita-Lumsden A., Harris P., Bower M. (2010). Lymphoma in the immunocompromised. Br. J. Hosp. Med..

[32] Thieblemont C., Coiffier B. (2007). Lymphoma in older patients. J. Clin. Oncol..

[33] De Antoni D.J., Claro M.L., Poehling G.G., Hughes S.S. (1996). Translaminar lumbar epidural endoscopy: Anatomy, technique, and indications. Arthroscopy.

[34] Lin G.X., Huang P., Kotheeranurak V., Park C.W., Heo D.H., Park C.K., Park J.Y., Kim J.S. (2019). A systematic review of unilateral biportal endoscopic spinal surgery: Preliminary clinical results and complications. World Neurosurg..

[35] Heo D.H., Quillo-Olvera J., Park C.K. (2018). Can percutaneous biportal endoscopic surgery achieve enough canal decompression for degenerative lumbar stenosis? Prospective case-control study. World Neurosurg..

[36] Kang M.S., Heo D.H., Kim H.B., Chung H.T. (2021). Biportal endoscopic technique for transforaminal lumbar interbody fusion: Review of current research. Int. J. Spine Surg..

[37] Heo D.H., Son S.K., Eum J.H., Park C.K. (2017). Fully endoscopic lumbar interbody fusion using a percutaneous unilateral biportal endoscopic technique: Technical note and preliminary clinical results. Neurosurg. Focus.

[38] Park J.H., Jun S.G., Jung J.T., Lee S.J. (2017). Posterior percutaneous endoscopic cervical foraminotomy and diskectomy with unilateral biportal endoscopy. Orthopedics.

[39] Kim J., Heo D.H., Lee D.C., Chung H.T. (2021). Biportal endoscopic unilateral laminotomy with bilateral decompression for the treatment of cervical spondylotic myelopathy. Acta Neurochir..

[40] Jing X., Gong Z., Qiu X., Zhong Z., Ping Z., Hu Q. (2022). “Cave-in” decompression under unilateral biportal endoscopy in a patient with upper thoracic ossification of posterior longitudinal ligament: Case report. Front. Surg..

[41] Kim J.Y., Ha J.S., Lee J.S., Lee C.K., Hong H.J., Choi S.Y., Park C.K. (2023). Biportal endoscopic posterior thoracic laminectomy for thoracic spondylotic myelopathy caused by ossification of the ligamentum flavum: Technical developments and outcomes. Neurospine.

[42] Kim S.K., Bendardaf R., Ali M., Kim H.A., Heo E.J., Lee S.C. (2022). Unilateral biportal endoscopic tumor removal and percutaneous stabilization for extradural tumors: Technical case report and literature review. Front. Surg..

[43] Peng W., Zhuang Y., Cui W., Chen W., Chu R., Sun Z., Zhang S. (2024). Unilateral biportal endoscopy for the resection of thoracic intradural extramedullary tumors: Technique case report and literature review. Int. Med. Case Rep. J..

